# Bacterial, Fungal, and Parasitic Zoonoses

**DOI:** 10.3390/pathogens13010005

**Published:** 2023-12-19

**Authors:** Valentina Virginia Ebani, Francesca Mancianti

**Affiliations:** 1 Department of Veterinary Sciences, University of Pisa, Viale delle Piagge 2, 56124 Pisa, Italy; francesca.mancianti@unipi.it; 2 Centre for Climate Change Impact, University of Pisa, Via del Borghetto 80, 56124 Pisa, Italy; 3 Interdepartmental Research Center “Nutraceuticals and Food for Health”, University of Pisa, Via del Borghetto 80, 56124 Pisa, Italy

Zoonoses encompass several bacterial, parasitic, and mycotic diseases of domestic and free-living animals. In recent years, we have witnessed the emergence of infectious diseases that exhibit different etiologies in their possible hosts, which range from various animals to humans. Zoonotic agents can remain undetected, being present in an asymptomatic form in animal reservoirs, which enhances the threat to human beings. The One Health approach aims for an optimal outcome in terms of the One Health Triad, namely, healthy people and healthy animals in a healthy environment [1]. For this reason, knowledge of the etiology, epidemiology, environmental impact, pathogenic effects, diagnosis, control, and treatment of such diseases is a necessity.

Fifteen manuscripts were submitted for consideration for the Special Issue, and all of them were subject to the rigorous *Pathogens* review process. In total, ten research articles were ultimately accepted for publication and inclusion in this Special Issue.

Topics concerning bacterial zoonoses were explored in five studies.

One paper is a contribution on Chlamydiales infection in domestic ruminants. Jonker and Michel aimed to optimize and apply qPCR assays for the detection of Chlamydiales in the products of domestic ruminant abortion. The authors focus on the detection of members of the order Chlamydiales and the differentiation of *Chlamydia abortus, Chlamydia pecorum, Parachlamydia acanthamoeba,* and *Waddlia chondrophila*. The authors have developed a diagnostic tool that can effectively identify the specific causes of reproductive disorders in farm ruminants. This tool is useful in the timely identification of the etiologic agent responsible for these disorders. Additionally, the study found that many of the investigated chlamydial agents, which are potential zoonotic bacteria, were detected. This highlights the importance of implementing appropriate safety precautions when handling material from abortion in ruminants. Overall, this diagnostic tool not only aids the identification of reproductive disorders in farm ruminants, but also emphasizes the importance of handling such materials with caution to prevent the potential transmission of zoonotic bacteria.

Four contributions explore salmonellosis in humans and animals. *Salmonella* spp. infection is currently a severe threat to livestock, pet animals, and humans, in whom it can cause severe symptomatology. Salmonellosis is a major public health concern worldwide and has great negative economic impacts due to the cost of the surveillance, investigation, treatment, and prevention of illness both in animals and people [2].

Murray et al. showed relevant *S. enterica* prevalences in poultry flocks in Ontario between 2009 and 2018; they found overall values of 25.3% in broilers, 6.4% in layers, and 28.6% in turkey breeders. These data confirm that salmonellae, despite preventive measures, are often present in poultry flocks, which is a concerning situation given that most cases of salmonellosis in humans are associated with the consumption of contaminated poultry products, such as meat or eggs [2].

Companion animals were shown to act as reservoirs of salmonellae and sources of infection for their owners in previous several investigations. *Salmonella*-infected pets are usually asymptomatic carriers, and they can shed one or more serotypes intermittently over several weeks. In recent years, reptiles have become common pet animals [3], and they have been frequently been found to be carriers of, and to excrete, different pathogens, including salmonellae [4]. Dec et al. confirmed this trend in pet snakes, lizards, and turtles in Poland, with an overall 71.6% prevalence; different serotypes belonging to the subspecies *enterica*, *arizonae, diarizonae,* and *salamae* were cultured. 

*Salmonella* strains of human and animal origin are often antibiotic-resistant. Resistance to fluoroquinolones (FQs) in Salmonella has been increasing worldwide, with WHO considering FQ-resistant *Salmonella* spp. as high-priority pathogens [5]. Piekarska et al. analyzed 447 *Salmonella* strains, isolated from humans in the years 2018–2019 in Poland, that belonged to the most common serotypes; 37.6% of the strains were shown to be FQ-resistant, and molecular analyses detected the corresponding resistance genes. 

Rahman et al. have developed a multiplex PCR test to investigate and distinguish *Salmonella* serotypes in a single PCR tube. The newly developed multiplex PCR appears to be a novel, simple, and reliable method of identifying six widely found *S. enterica* subsp. *enterica* strains, including typhoidal and nontyphoidal serotypes. This test is a rapid and high-quality single-nucleotide-polymorphism (SNP)-based method that is useful for improving the detection and identification of salmonellae; therefore, it could facilitate the timely prevention and treatment of this important zoonosis.

Parasitological topics are addressed in two epidemiological studies on *Cryptosporidium* spp. These Apicomplexan protozoa are responsible for acute gastroenteritis, abdominal pain, and diarrhea. The genus encompasses several zoonotic or potentially zoonotic species, including, firstly, *Cryptosporidium parvum.* These protozoa are recognized as a cause of infant malnutrition, frequently leading to the premature death of children under five years of age [6]; however, this parasitosis is underdiagnosed and underestimated [7]. Cryptosporidia are transmitted mostly via the fecal–oral route and, less frequently, via inhalation when the respiratory system is involved [8]. Due to the small size of the oocysts, the parasite spreads via water.

In this Special Issue, the zoonotic transmission of *Cryptosporidium canis* among children and their pets, and the probable transmission of *C. parvum* among four children and their cats and dogs, were investigated by Coehlo et al., and they shed light on some lesser-known epidemiologic features. Similarly, Panegossi et al. conducted a study on the prevalence of *Cryptosporidium proventriculi* infection in captive *Nymphicus hollandicus,* where gastrointestinal signs were considered significant predictors of infection. Although this has not been proven to be a zoonotic species, the authors do not rule out potential risks to bird owners.

*Giardia duodenalis* is a flagellate protozoan infecting the upper intestinal tract in humans and animals worldwide. Transmission occurs via the fecal–oral route and includes direct contact or the contamination of water and food [9]. Assemblages A and B are well recognized as potentially zoonotic, but assemblages C, D, E, F, and G have also been identified in human infections, suggesting a wider zoonotic potential for the parasite [10]. In one such scenario, Maestrini et al. reported and discussed the occurrence and molecular characterization of assemblage A and mixed assemblages A and B from 18/43 badgers (*Meles meles*) from Italy. Sub-assemblage AII, a human-specific one, was also identified, corroborating a role of this animal species in spreading potentially zoonotic parasites from latrines to human settlements.

Toxocariasis is a widespread human helminth zoonosis caused by the infective larval stages of *Toxocara* spp. from the environment. *Toxocara canis* and, to a lesser extent, *Toxocara cati* are the main species responsible for “Larva migrans syndrome”, which clinically develops in generalized, neurological, ocular, covert, and asymptomatic forms [11]. Toxocariasis is reported worldwide as a neglected parasite disease, to which about a fifth of the world population is exposed [12]. Treatment is a challenge due to the difficulty of verifying drug efficacy in patients, but albendazole is preferable because it is widely distributed throughout tissues when metabolized. This statement is confirmed by Magnaval et al., who report the results of a retrospective study comparing the efficacy of Diethylcarbamazine and Albendazole on a cohort of patients affected by toxocariasis. Albendazole at a 10–15 mg/kg b/w daily dose over a 2-week course was demonstrated to be the treatment of choice for human toxocariasis, showing few adverse effects. Diethylcarbamazine is suggested as an alternative option for patients for whom Albendazole therapy has failed.

Filariid helminths are transmitted via blood-sucking insects, and those affecting equids have scarcely been investigated; thus, the equine diseases for which they are responsible have been neglected. The paper by Abo-Aziza et al. refers to the occurrence of *Setaria digitata, Dirofilaria repens*, and *Mansonella* sp. in horses and donkeys from Egypt. The authors administered a dosage of circulating cytokines to evaluate the response from the infected host. Animals infected with *S. digitata* displayed a Th1 immune responses in contrast to equines positive for *Mansonella* sp. 

Zoonoses continue to be a significant concern in both human and veterinary medicine. Despite the implementation of preventive measures such as maintaining hygiene in the environment, controlling farm and companion animals, and vaccination, zoonoses remain a relevant topic. This Special Issue features articles that provide further evidence of the ongoing interest in zoonoses and the need for continued research and efforts to control and prevent these diseases.

The contributions of the Special Issue are chronologically listed below.
